# Retraction Note: The impact of repeated vaccination on influenza vaccine effectiveness: a systematic review and meta-analysis

**DOI:** 10.1186/s12916-018-1137-0

**Published:** 2018-08-12

**Authors:** Lauren C. Ramsay, Sarah A. Buchan, Robert G. Stirling, Benjamin J. Cowling, Shou Feng, Jeffrey C. Kwong, Bryna F. Warshawsky

**Affiliations:** 1 Public Health Ontario, 480 University Avenue Suite 300, Toronto, ON M5G 1V2 Canada; 2 Dalla Lana School of Public Health, University of Toronto, 155 College St, Toronto, ON M5T 3M7 Canada; 3 Public Health Agency of Canada, 130 Colonnade Road, Ottawa, ON K1A 0K9 Canada; 4 WHO Collaborating Centre for Infectious Disease Epidemiology and Control, School of Public Health, Li Ka Shing Faculty of Medicine, The University of Hong Kong, Pokfulam, Hong Kong, China; 5 Institute for Clinical Evaluative Sciences, Veterans Hill Trail, 2075 Bayview Avenue G1 06, Toronto, ON M4N 3M5 Canada; 6 Department of Family & Community Medicine, University of Toronto, 155 College St, Toronto, ON M5T 3M7 Canada; 7 University Health Network, 399 Bathurst St, Toronto, ON M5T 2S8 Canada; 8 Public Health Ontario, 480 University Avenue Suite 300, Toronto, ON M5G 1V2 Canada; 9 Department of Epidemiology and Biostatistics, Western University, 1151 Richmond St, London, ON N6A 3K7 Canada

## Retraction Note

The authors have retracted this article, *The impact of repeated vaccination on influenza vaccine effectiveness: a systematic review and meta-analysis*. Upon reviewing the published work, the authors determined that there were a few erroneous inclusions and an omission of estimates which impacted the conclusion for the influenza A(H3N2) and influenza B analyses comparing vaccination in both years to the current year only. All authors agree to this retraction, and they have been given the opportunity to submit a revised version in the journal.

## References

[1] Ramsay LC (2017). The impact of repeated vaccination on influenza vaccine effectiveness: a systematic review and meta-analysis. BMC Med.

